# Supplementary material for: Within-individual variation of C-reactive protein (CRP) measurements in primary care: A retrospective cohort study
Source: PLoS One. 2025 Nov 24;20(11):e0337221. doi: 10.1371/journal.pone.0337221 (PMC12643293; doi:10.1371/journal.pone.0337221)
Supplement: S1 File — (DOCX) [file pone.0337221.s001.docx]

Appendix 1 - Subgroup analyses

**Table S1 Other subgroup analyses.** *Total N=472,811*

|  |  | N | % | Median (mg/mL) | Log adj CV (95%CI) | 95% CI |
| --- | --- | --- | --- | --- | --- | --- |
| Comorbidities | No comorbidity | 125,268 | 26.5 | 4.0 | 1.715 | 1.710 - 1.719 |
|  | Hypertension | 160,436 | 33.9 | 5.8 | 1.461 | 1.458 - 1.464 |
|  | Asthma | 93,709 | 19.8 | 5.0 | 1.397 | 1.393 - 1.401 |
|  | COPD | 35,429 | 7.5 | 8.0 | 1.451 | 1.445 - 1.458 |
|  | Hyperthyroidism | 10,580 | 2.2 | 5.0 | 1.430 | 1.418 - 1.441 |
|  | Hypothyroidism | 50,948 | 10.8 | 5.0 | 1.442 | 1.437 - 1.447 |
|  | CKDstage3 to 5 | 54,173 | 11.5 | 6.0 | 1.537 | 1.531 - 1.542 |
|  | Heart failure | 12,732 | 2.7 | 8.0 | 1.317 | 1.307 -1.327 |
|  | Heart disease | 52,157 | 11.0 | 5.2 | 1.508 | 1.503 - 1.514 |
|  | Ischaemic stroke | 6,783 | 1.4 | 6.0 | 1.560 | 1.543 - 1.577 |
|  | Haem. Stroke | 2,453 | 0.5 | 6.0 | 1.676 | 1.645 - 1.707 |
|  | Cancer | 38,658 | 8.2 | 5.6 | 1.579 | 1.572 - 1.586 |
|  | PreDM or DM | 5,967 | 1.3 | 5.0 | 1.201 | 1.189 - 1.213 |
| BMI | ≤18.5 | 8,633 | 1.8 | 4.3 | 1.927 | 1.906 - 1.948 |
|  | >18.5 to 25 | 136,643 | 28.9 | 4.6 | 1.762 | 1.758 - 1.766 |
|  | >25 to 30 | 129,782 | 27.4 | 5.0 | 1.652 | 1.648 - 1.656 |
|  | >30 to 35 | 60,450 | 12.8 | 6.0 | 1.532 | 1.527 - 1.537 |
|  | >35 | 137,303 | 29.0 | 5.0 | 1.415 | 1.412 - 1.419 |
| Region | East Midlands | 2,734 | 0.6 | 0.6 | 2.014 | 1.971 - 2.058 |
|  | East of England | 17,098 | 3.6 | 3.6 | 1.055 | 1.049 - 1.060 |
|  | London | 30,912 | 6.5 | 6.5 | 1.281 | 1.274 - 1.287 |
|  | North East | 2,505 | 0.5 | 0.5 | 0.986 | 0.975 - 0.997 |
|  | North West | 42,535 | 9.0 | 9.0 | 1.157 | 1.153 - 1.161 |
|  | Northern Ireland | 58,593 | 12.4 | 12.4 | 4.519 | 4.492 - 4.547 |
|  | Scotland | 66,764 | 14.1 | 14.1 | 1.158 | 1.154 - 1.161 |
|  | South Central | 54,237 | 11.5 | 11.5 | 1.542 | 1.537 - 1.547 |
|  | South East Coast | 46,868 | 9.9 | 9.9 | 1.510 | 1.504 - 1.516 |
|  | South West | 40,816 | 8.6 | 8.6 | 1.436 | 1.430 - 1.442 |
|  | Wales | 82,139 | 17.4 | 17.4 | 1.070 | 1.067 - 1.073 |
|  | West Midlands | 24,523 | 5.2 | 5.2 | 1.294 | 1.288 - 1.301 |
|  | Yorkshire & Humber | 3,087 | 0.7 | 0.7 | 1.128 | 1.113 - 1.143 |
| Alcohol consumption | Current drinker | 273,066 | 57.8 | 5.0 | 1.614 | 1.611 - 1.617 |
|  | Ex drinker | 6,654 | 1.4 | 5.0 | 1.781 | 1.775 - 1.787 |
|  | Teetotal | 85,921 | 18.2 | 5.0 | 1.460 | 1.456 - 1.464 |
|  | Missing | 107170 | 22.7 | - | - | - |
| Smoker Status | Current Smoker | 99,438 | 21.0 | 5.0 | 1.460 | 1.456 - 1.464 |
|  | Ex-smoker | 87,712 | 18.6 | 5.0 | 1.684 | 1.679 – 1.689 |
|  | Never smoked | 222,482 | 47.1 | 5.0 | 1.724 | 1.721 – 1.728 |
|  | Missing | 63179 | 13.4 | - | - | - |

**Figure S1 CVT by region**

**
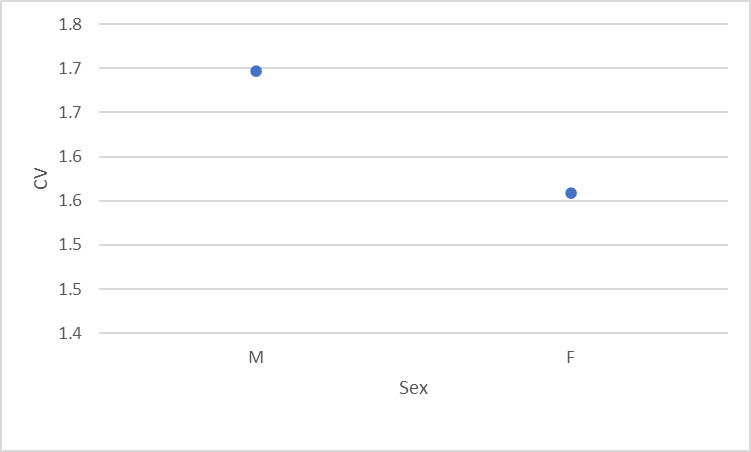
**

**Figure S2 CVT by sex**

**
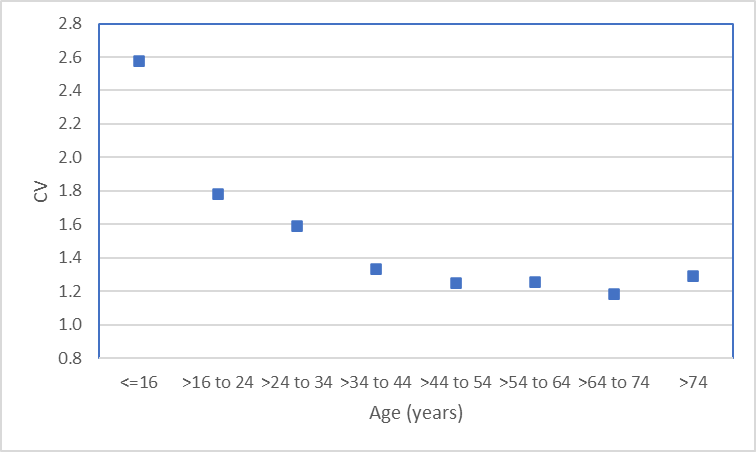
**

**Figure S3 CVT by age**

**
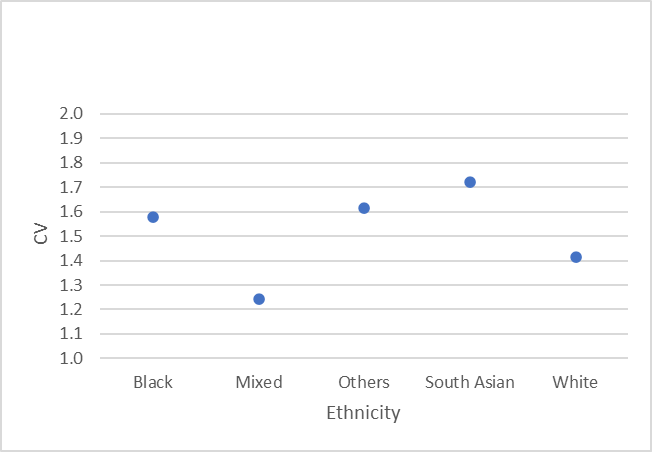
**

**Figure S4 CVT by ethnicity**

**
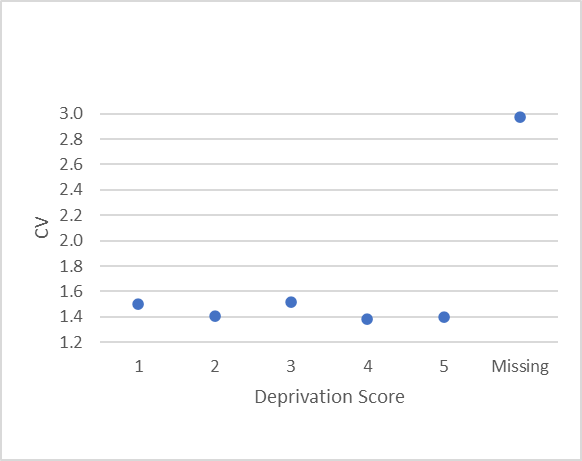
**

**Figure S5 CVT by Townsend deprivation score**


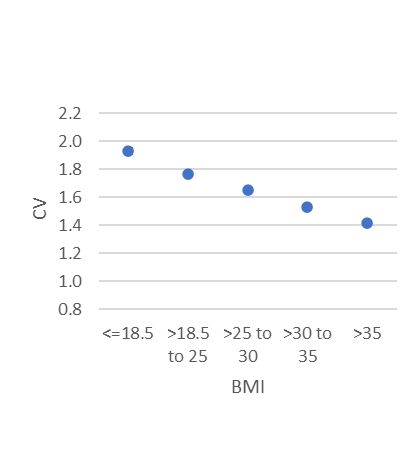


**Figure S6 CVT by BMI**

**
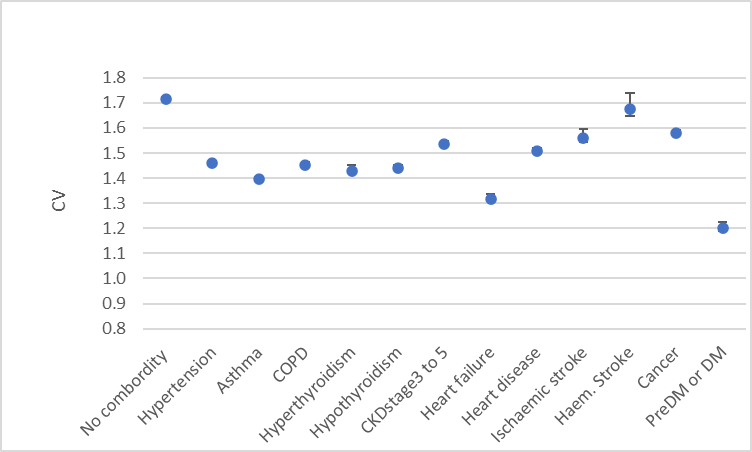
**

**Figure S7 CVT by comorbidities**

**
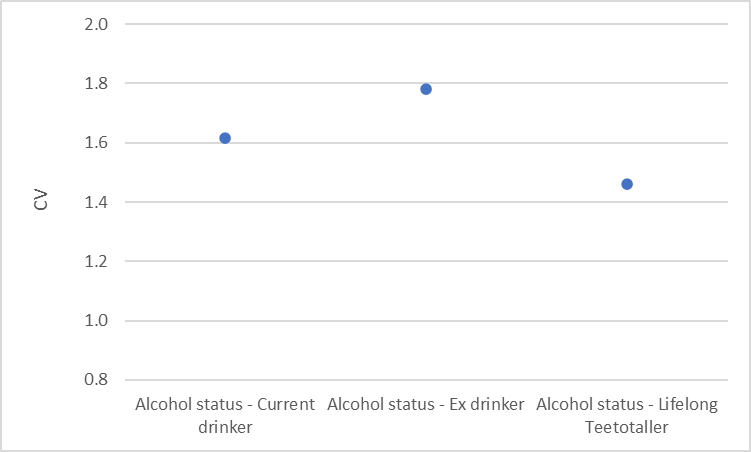
**

**Figure S8 CVT by alcohol consumption**

**Figure S9 CVT by smoker status**

Appendix 2 – sensitivity analyses

**Table S2 Sensitivity analyses**

| *Total N=472,811* |  | N | % | Median (mg/L) | CV | 95% CIs |
| --- | --- | --- | --- | --- | --- | --- |
| Year | 2010 | 14,663 | N/A | 6.0 | 0.831 | 1.148 - 1.164 |
|  | 2011 | 17,219 | N/A | 5.8 | 0.883 | 1.090 - 1.103 |
|  | 2012 | 19,028 | N/A | 5.0 | 0.884 | 1.135 - 1.148 |
|  | 2013 | 20,329 | N/A | 5.0 | 0.920 | 0.964 - 0.974 |
|  | 2014 | 20,667 | N/A | 5.0 | 0.925 | 0.974 - 0.985 |
|  | 2015 | 19,257 | N/A | 5.0 | 0.899 | 0.933 - 0.943 |
|  | 2016 | 15,867 | N/A | 5.0 | 0.938 | 0.954 - 0.966 |
|  | 2017 | 14,397 | N/A | 5.0 | 0.907 | 0.989 - 1.002 |
|  | 2018 | 13,920 | N/A | 5.0 | 0.958 | 1.049 - 1.064 |
|  | 2019 | 13,533 | N/A | 6.0 | 0.953 | 0.946 - 0.960 |
| Number of measurements | 2 | 1,077 | N/A | 9.0 | 2.650 | 2.404 - 2.940 |
|  | 3 | 2,465 | N/A | 7.0 | 2.306 | 2.210 - 2.409 |
|  | 4 | 146,977 | N/A | 5.0 | 1.777 | 1.770 - 1.784 |
|  | 5 | 90,746 | N/A | 5.0 | 1.722 | 1.715 - 1.730 |
|  | 6 | 59,023 | N/A | 5.0 | 1.715 | 1.707 - 1.723 |
|  | 7 | 39,131 | N/A | 5.0 | 1.743 | 1.734 - 1.752 |
|  | 8 | 26,919 | N/A | 5.0 | 1.699 | 1.689 - 1.709 |
|  | 9 | 19,107 | N/A | 5.0 | 1.739 | 1.728 - 1.751 |
|  | 10 to 15 | 48,151 | N/A | 5.0 | 1.668 | 1.662 - 1.674 |
|  | 16 to 20 | 13,190 | N/A | 5.0 | 1.461 | 1.454 - 1.468 |
|  | 21 to 25 | 6,925 | N/A | 5.0 | 1.465 | 1.457 - 1.474 |
|  | 26 to 30 | 4,547 | N/A | 5.0 | 1.404 | 1.395 - 1.413 |
|  | 31 to 40 | 5,674 | N/A | 5.0 | 1.310 | 1.304 - 1.317 |
|  | 41 to 50 | 3,578 | N/A | 5.0 | 1.329 | 1.322 - 1.336 |
|  | 51 to 60 | 2,357 | N/A | 5.0 | 1.170 | 1.163 - 1.177 |
|  | 61 to 70 | 1,674 | N/A | 5.0 | 1.468 | 1.458 - 1.479 |
|  | 71 to 80 | 1,225 | N/A | 5.5 | 1.309 | 1.300 - 1.319 |
|  | 81 to 90 | 910 | N/A | 5.0 | 1.682 | 1.667 - 1.697 |
|  | 91 to 100 | 618 | N/A | 5.1 | 1.889 | 1.868 - 1.910 |
|  | >100 | 1,817 | N/A | 6.0 | 1.649 | 1.641 - 1.658 |
| Patient mean CRP (mg/L) | 0 to <1 | 9,070 | 1.9 | 0.3 | 11.036 | 10.706 - 11.381 |
|  | 1 to < 2 | 36,943 | 7.8 | 1.0 | 1.893 | 1.882 - 1.903 |
|  | 2 to <3 | 40,888 | 8.6 | 2.0 | 1.596 | 1.588 - 1.603 |
|  | 3 to < 4 | 41,919 | 8.9 | 3.0 | 1.359 | 1.354 - 1.365 |
|  | 4 to <5 | 38,632 | 8.2 | 4.0 | 1.298 | 1.293 - 1.303 |
|  | 5 to <6 | 32,433 | 6.9 | 5.0 | 1.157 | 1.153 - 1.162 |
|  | 6 to < 7 | 26,634 | 5.6 | 5.0 | 1.234 | 1.229 - 1.240 |
|  | 7 to < 8 | 22,522 | 4.8 | 5.0 | 1.356 | 1.350 - 1.363 |
|  | 8 to < 9 | 19,124 | 4.0 | 6.0 | 1.292 | 1.285 - 1.298 |
|  | 9 to < 10 | 16,760 | 3.5 | 6.1 | 1.296 | 1.289 - 1.303 |
|  | 10 to <20 | 93,199 | 19.7 | 9.0 | 1.481 | 1.477 - 1.485 |
|  | 20 to < 30 | 39,105 | 8.3 | 14.6 | 1.757 | 1.749 - 1.764 |
|  | 30 to < 40 | 20,727 | 4.4 | 20.0 | 2.101 | 2.086 - 2.115 |
|  | 40 to < 50 | 11,923 | 2.5 | 26.0 | 2.314 | 2.290 - 2.337 |
|  | 50 to < 60 | 7,619 | 1.6 | 32.0 | 2.528 | 2.494 - 2.564 |
|  | 60 to < 70 | 4,869 | 1.0 | 40.0 | 2.882 | 2.827 - 2.938 |
|  | 70 to < 80 | 3,378 | 0.7 | 48.0 | 2.860 | 2.794 - 2.929 |
|  | 80 to < 90 | 2,129 | 0.5 | 58.4 | 2.855 | 2.772 - 2.942 |
|  | 90 to < 100 | 1,444 | 0.3 | 68.0 | 2.823 | 2.723 - 2.928 |
|  | 100 to < 200 | 3,367 | 0.7 | 104.0 | 2.971 | 2.898 - 3.048 |
|  | 200 to < 300 | 119 | 0.0 | 235.4 | 1.848 | 1.666 - 2.064 |
|  | =>300 | 7 | 0.0 | 323.0 | 1.243 | 0.883 - 1.883 |
| Days between measurements | 0 to < 4 | 908 | 0.2 | 36.6 | 1.921 | 1.852 - 1.994 |
|  | 4 to < 7 | 1,387 | 0.3 | 22.0 | 2.024 | 1.958 - 2.093 |
|  | 7 to < 14 | 6,771 | 1.4 | 12.2 | 1.621 | 1.603 - 1.639 |
|  | 14 to < 21 | 8,970 | 1.9 | 9.0 | 1.447 | 1.437 - 1.457 |
|  | 21 to < 28 | 8,164 | 1.7 | 8.8 | 1.561 | 1.549 - 1.574 |
|  | 28 to < 35 | 19,412 | 4.1 | 6.0 | 1.294 | 1.291 - 1.298 |
|  | 35 to < 42 | 10,769 | 2.3 | 6.0 | 1.681 | 1.671 - 1.690 |
|  | 42 to < 49 | 9,182 | 1.9 | 6.0 | 1.600 | 1.590 - 1.610 |
|  | 49 to 60 | 13,993 | 3.0 | 6.0 | 1.489 | 1.481 - 1.496 |
|  | 60 to < 90 | 28,334 | 6.0 | 6.0 | 1.464 | 1.458 - 1.470 |
|  | 90 to < 120 | 25,126 | 5.3 | 5.1 | 1.564 | 1.556 - 1.572 |
|  | 120 to < 150 | 21,156 | 4.5 | 5.0 | 1.648 | 1.638 - 1.659 |
|  | 150 to < 180 | 20,733 | 4.4 | 5.0 | 1.658 | 1.647 - 1.669 |
|  | 180 to < 270 | 58,359 | 12.3 | 5.0 | 1.655 | 1.648 - 1.662 |
|  | 270 to < 365 | 54,672 | 11.6 | 4.0 | 1.667 | 1.659 - 1.675 |
|  | 365 to < 455 | 41,855 | 8.9 | 4.0 | 1.698 | 1.688 - 1.707 |
|  | 455 to < 545 | 32,227 | 6.8 | 4.0 | 1.775 | 1.763 - 1.787 |
|  | 545 to < 635 | 25,200 | 5.3 | 4.0 | 1.982 | 1.966 - 1.999 |
|  | 635 to < 730 | 20,949 | 4.4 | 4.0 | 1.887 | 1.869 - 1.904 |
|  | >730 | 64,526 | 13.6 | 4.0 | 2.226 | 2.213 - 2.240 |
| Method | Linear regression (log adjusted data) | 472,811 | 100 | 5.0 | 1.604 | 1.602 to 1.606 |
|  | Linear regression (non log-adjusted data) | 472,811 | 100 | 5.0 | 1.789 | 1.782 – 1.795 |
|  | Arithmetic method | 472,811 | 100 | 5.0 | 0.998 | 0.005 - 5.869 (range) |

**
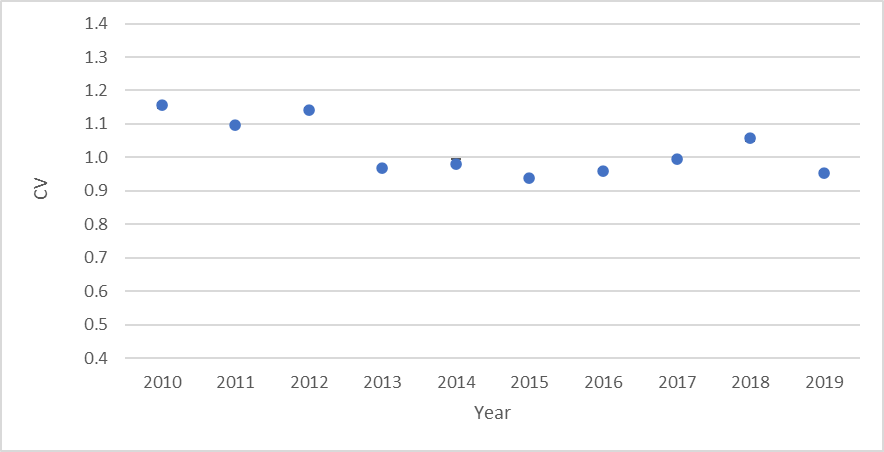
**

**Figure S10 CVT by year**

**Table S3 CVT by quarter**

| Year | Q1 | Q2 | Q3 | Q4 |
| --- | --- | --- | --- | --- |
| 2010 | 1.297 (1.257-1.339) | 1.182 (1.148-1.218) | 1.187 (1.154-1.221) | 1.089 (1.059-1.119) |
| 2011 | 1.063 (1.036-1.091) | 0.883 (0.862-0.904) | 0.935 (0.914-0.956) | 0.868 (0.848-0.889) |
| 2012 | 1.093 (1.067-1.119) | 1.065 (1.039-1.091) | 1.230 (1.199-1.263) | 0.891 (0.871-0.911) |
| 2013 | 0.917 (0.897-0.939) | 0.967 (0.945-0.989) | 0.916 (0.897-0.937) | 0.888 (0.868-0.908) |
| 2014 | 0.974 (0.952-0.997) | 1.000 (0.977-1.024) | 0.905 (0.885-0.924) | 0.947 (0.926-0.970) |
| 2015 | 1.016 (0.992-1.041) | 0.869 (0.850-0.889) | 0.856 (0.837-0.875) | 0.869 (0.848-0.890) |
| 2016 | 1.034 (1.008-1.061) | 0.957 (0.933-0.981) | 0.974 (0.948-1.000) | 0.945 (0.919-0.973) |
| 2017 | 1.150 (1.117-1.184) | 0.965 (0.938-0.993) | 0.918 (0.892-0.944) | 0.944 (0.916-0.973) |
| 2018 | 1.184 (1.147-1.223) | 1.041 (1.010-1.074) | 0.994 (0.964-1.024) | 1.157 (1.120-1.196) |
| 2019 | 0.988 (0.959-1.019) | 0.924 (0.897-0.952) | 0.932 (0.906-0.959) | 0.899 (0.874-0.926) |

| Year | Q1 | Q2 | Q3 | Q4 |
| --- | --- | --- | --- | --- |
| 2010 | 1.297 (1.257-1.339) | 1.182 (1.148-1.218) | 1.187 (1.154-1.221) | 1.089 (1.059-1.119) |
| 2011 | 1.063 (1.036-1.091) | 0.883 (0.862-0.904) | 0.935 (0.914-0.956) | 0.868 (0.848-0.889) |
| 2012 | 1.093 (1.067-1.119) | 1.065 (1.039-1.091) | 1.230 (1.199-1.263) | 0.891 (0.871-0.911) |
| 2013 | 0.917 (0.897-0.939) | 0.967 (0.945-0.989) | 0.916 (0.897-0.937) | 0.888 (0.868-0.908) |
| 2014 | 0.974 (0.952-0.997) | 1.000 (0.977-1.024) | 0.905 (0.885-0.924) | 0.947 (0.926-0.970) |
| 2015 | 1.016 (0.992-1.041) | 0.869 (0.850-0.889) | 0.856 (0.837-0.875) | 0.869 (0.848-0.890) |
| 2016 | 1.034 (1.008-1.061) | 0.957 (0.933-0.981) | 0.974 (0.948-1.000) | 0.945 (0.919-0.973) |
| 2017 | 1.150 (1.117-1.184) | 0.965 (0.938-0.993) | 0.918 (0.892-0.944) | 0.944 (0.916-0.973) |
| 2018 | 1.184 (1.147-1.223) | 1.041 (1.010-1.074) | 0.994 (0.964-1.024) | 1.157 (1.120-1.196) |
| 2019 | 0.988 (0.959-1.019) | 0.924 (0.897-0.952) | 0.932 (0.906-0.959) | 0.899 (0.874-0.926) |

**
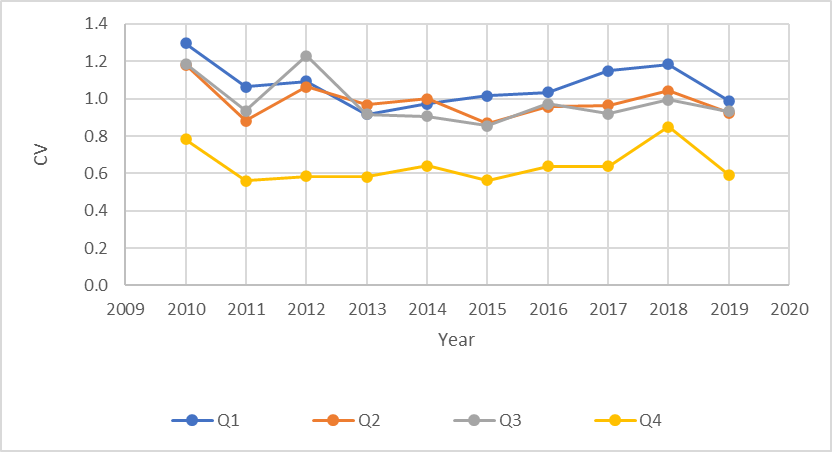
**

**Figure S11 CVT by quarter**

**
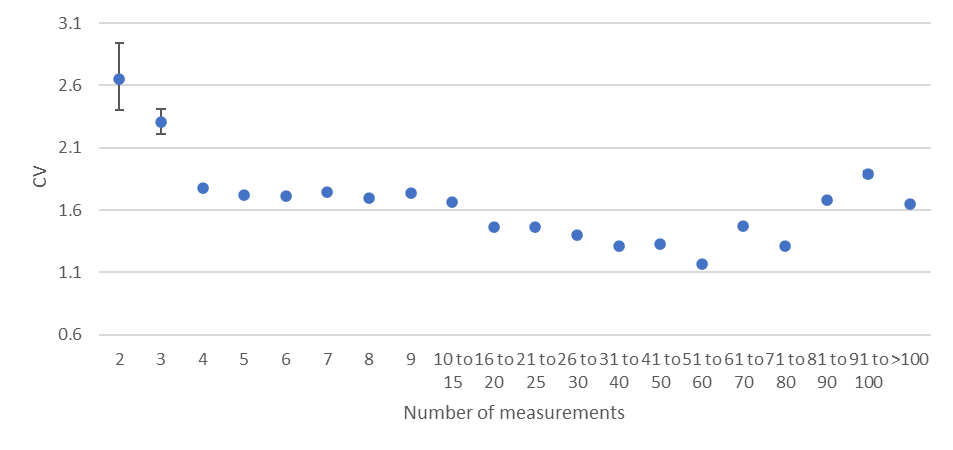
**

**Figure S12 CVT by Number of measurements**

**
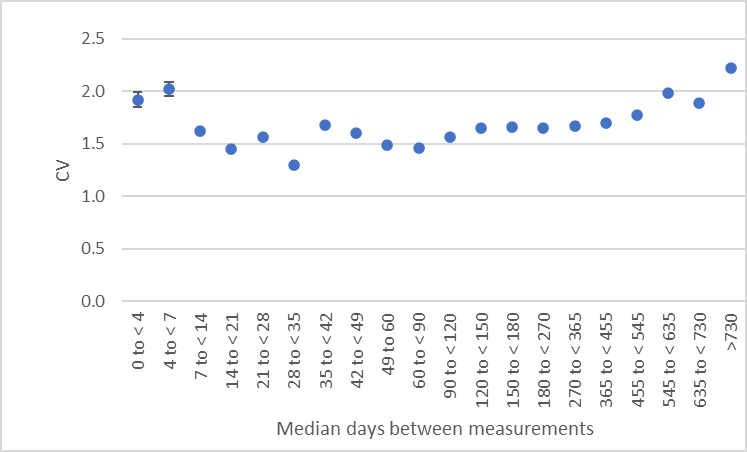
**

**Figure S13 CVT by days between measurements**

**
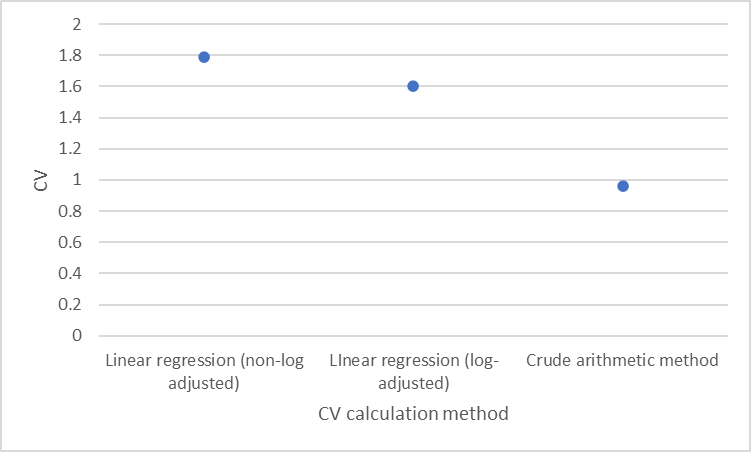
**

**Figure S14** **CVT by arithmetic calculation methods (using non-log adjusted data ) and linear regression (using non-log adjusted data)**

**
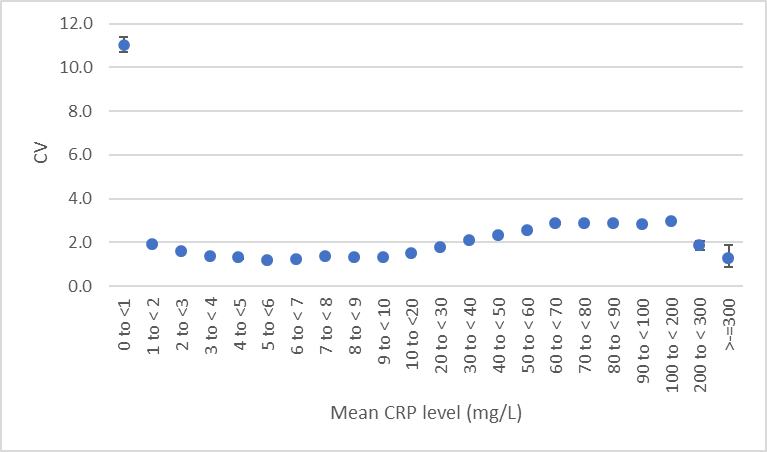
**

**Figure S15** **CVT by patient mean**
